# PTEN-induced kinase 1 enhances the reparative effects of bone marrow mesenchymal stromal cells on mice with renal ischaemia/reperfusion-induced acute kidney injury

**DOI:** 10.1007/s13577-022-00756-8

**Published:** 2022-08-12

**Authors:** Chenyu Lin, Wen Chen, Yong Han, Yujie Sun, Xiaoqiong Zhao, Yuan Yue, Binyu Li, Wenmei Fan, Tao Zhang, Li Xiao

**Affiliations:** 1 Institute of Respiratory and Critical Medicine, Beijing Key Laboratory of Organ Transplantation and Immunology Regulatory, the 8th Medical Centre of Chinese PLA General Hospital, No. 17 Heishan Hu road, Qinglongqiao street, Haidian district, Beijing, 100091 China; 2 Jiamusi University, Jiamusi, China

**Keywords:** PTEN-induced kinase 1 (PINK1), Bone marrow mesenchymal stromal cell, Ischaemia/reperfusion injury, Acute kidney injury

## Abstract

Acute kidney injury (AKI) is a common severe acute syndrome caused by multiple factors and is characterized by a rapid decline in renal function during a short period. Bone marrow mesenchymal stromal cells (BMSCs) are effective in treating AKI. However, the mechanism of their beneficial effects remains unclear. PTEN-induced kinase 1 (PINK1) may play an important role in kidney tissue repair. In this study, we explored the effect of PINK1 overexpression on enhancing BMSC-mediated repair of AKI. In this study, ischaemia/reperfusion-induced AKI (IRI-AKI) in mice and a hypoxia-reoxygenation model in cells were established, and the indices were examined by pathology and immunology experiments. After ischaemia/reperfusion, PINK1 overexpression reduced apoptosis in injured kidney tissue cell, decreased T lymphocyte infiltration, increased macrophage infiltration, and alleviated the inflammatory response. PINK1 relieved the stress response of BMSCs and renal tubular epithelial cells (RTECs), reduced apoptosis, altered the release of inflammatory factors, and reduced the proliferation of peripheral blood mononuclear cells (PBMCs). In conclusion, BMSCs and RTECs undergo stress responses in response to hypoxia, inflammation and other conditions, and overexpressing PINK1 in BMSCs could enhance their ability to resist these stress reactions. Furthermore, PINK1 overexpression can regulate the distribution of immune cells and improve the inflammatory response. The regulation of mitochondrial autophagy during IRI-AKI maintains mitochondrial homeostasis and protects renal function. The results of this study provide new strategies and experimental evidence for BMSC-mediated repair of IRI-AKI.

## Introduction

Acute kidney injury (AKI) is a severe acute syndrome caused by multiple factors and is characterized by a rapid decline in renal function. AKI has become a serious disease that affects human health and longevity due to its high incidence, high mortality and poor prognosis [1, 2]. The leading causes of AKI include sepsis, nephrotoxin, and renal ischaemia–reperfusion injury (IRI) [3]. IRI is the main cause of AKI. In clinical practice, hypovolemia, hypotension or heart failure can cause transient ischaemia, which can further induce AKI. Acute tubular necrosis caused by IRI, especially early graft dysfunction and enhanced immunogenicity caused by IRI during kidney transplant, seriously affects the prognosis of patients [4]. There is a lack of effective treatments for AKI. Symptomatic treatment, supportive treatment or renal replacement therapy is usually used in clinical practice to eliminate aetiology and correct reversible risk factors. Currently, there is no specific treatment for AKI. A more thorough understanding of the basic molecular and immunological mechanisms underlying this complex condition would be conducive to identifying a starting point for therapeutic interventions.

The pathophysiological process of renal ischaemia/reperfusion-induced acute kidney injury (IRI-AKI) is complex. Substantial evidence demonstrates that mitophagy is the key link in the development of AKI. IRI-induced AKI is closely related to changes in mitochondrial function. Injured mitochondria can trigger apoptosis in renal tubular cells, activate the NOD-like receptor protein 3 (NLRP3) inflammasome, and eventually cause the loss of renal function [5]. One of the key adaptive responses of mitochondria to various stress-associated injuries is to activate mitophagy. During this process, injured mitochondria are rapidly and selectively removed in an autophagy-dependent manner, thus reducing the excessive release of reactive oxygen species (ROS) and proapoptotic substances caused by injured mitochondria. This mechanism is closely related to apoptosis, tissue remodelling and fibrosis. Therefore, maintaining mitochondrial homeostasis and function by promoting mitophagy may improve ischaemic kidney injury [6]. Mitophagy is mainly regulated by ubiquitin-dependent and ubiquitin-independent mechanisms, among which the ubiquitin-dependent pathway is the PTEN-induced putative kinase 1 (PINK1)/Parkin pathway. Recent reports suggest that the PINK1/Parkin pathway plays an important role in AKI-associated mitochondrial quality control, renal tubular cell survival and renal function protection [7]. PINK1 is the key enzyme associated with mitophagy, and it has protein kinase activity and can regulate cell function by phosphorylating various proteins in cells [8]. In addition, this enzyme can regulate mitochondrial morphology, function and autophagy through multiple pathways; PINK1 has an extremely low expression level when mitochondrial function is normal but a high expression level in high energy consumption tissues. It also has various biological activities and may participate in the occurrence, development and regulation of various diseases [9]. Kidney tissues with high energy consumption have abundant mitochondria, and mitochondrial damage or dysfunction is closely related to AKI caused by various factors. Therefore, the kinase PINK1 is a potential target for the treatment of AKI.

In recent years, stem cell therapy has become one of the most promising treatments for renal IRI-AKI, with greater potential value than single drug therapy due to the highly diverse responses of cells to the environment. Importantly, bone marrow mesenchymal stromal cells (BMSCs) have physiological activities in interstitial fibrosis, inflammation, angiogenesis and immune regulation. According to many studies, BMSCs are effective in various preclinical models of kidney injury or may improve the structure and function of the kidney after IRI by reducing inflammation and apoptosis, promoting autophagy, improving tissue fibrosis and accelerating the proliferation of renal interstitial cells through paracrine and endocrine factors [10, 11]. Although BMSCs have high therapeutic potential for kidney injuries, the transplantation and therapeutic effects of these cells are limited by the pathophysiological conditions of the injured site. Glucose deficiency, oxidative stress, ischaemia and inflammation induce BMSC dysfunction, affect the survival of transplanted BMSCs, and result in the death of transplanted cells in injured tissues [12, 13]. Among them, oxidative stress is the most unfavourable factor. Some studies revealed that mitochondrial dysfunction reduces the proliferation, migration and pluripotency of BMSCs [14, 15]. Therefore, to improve BMSC-mediated repair, it is critical to improve the survival rates of BMSCs transplanted in the injured site by improving the resistance of BMSCs under these pathophysiological conditions.

In this study, the key mitophagy enzyme PINK1 was induced by gene transfection and overexpressed in BMSCs to optimize the ability of these cells to resist damage under IRI-AKI conditions, which exerted effects on renal protection by improving the survival and differentiation of BMSCs under IRI-AKI conditions, regulating mitophagy and maintaining mitochondrial homeostasis, jointly enhancing BMSC-mediated repair of IRI-AKI, and then exerting a protective effect on renal function.

## Materials and methods

### Animals

Male C57BL/6 mice (6–8 weeks old, SPF grade) were purchased from SPF Biotechnology Co.(Beijing, China), Ltd. (animal licence NO. SCXK (Jing) 2019–0010). All experiments were approved by the Ethics Committee of Laboratory Animal Management in the Eighth Medical Center of Chinese PLA General Hospital (309,202,106,021,005) and were carried out according to the guidelines of The Basel Declaration and The International Council for Laboratory Animal Science.

### Cell culture

BMSCs from C57BL/6 mice and red fluorescent protein-labelled BMSCs (RFP-BMSCs) (MUBMX-01001, MUBMX-01201;Cyagen, Suzhou, China) were subjected to routine culture. After 24 h of resuscitation, the medium was changed for the first time, and the culture medium was changed every 2–3 days thereafter. The cells were passaged when they reached 80–90% confluence. Cells in the third generation were digested with 0.25% trypsin (Gibco, Grand Island, USA) containing 0.02% EDTA, washed twice with PBS (Hyclone, Logan, UT, USA), and then resuspended in PBS to make a live cell suspension with a concentration of 1 × 10^7^ cells/ml.

Renal tubular epithelial cells (RTECs) from C57BL/6 mice (CP-M062; Procell, Wuhan, China) were plated a 25 cm^2^ gelatine-coated culture flask containing C57BL/6 Mouse RTECs Complete Medium (CM-M062; Procell, Wuhan, China) in a water bath at 37 °C and placed in an incubator for routine incubation. Third-generation cells were resuspended in PBS and made into live cell suspensions at a concentration of 1 × 10^7^ cells/ml.

### PINK1 overexpression in BMSCs

The cells were digested and centrifuged when they reached 80%–90% confluence, and these cells were resuspended to the required concentration with antibiotic-free medium and inoculated into a 12-well plate (Corning, NY, USA) at a concentration of 0.5 × 10^4^ cells/ml. Subsequently, the plate was routinely cultured at 37 °C with 5% CO_2_ and 95% humidity, and antibiotic-free medium was replaced when the cells reached 60–70% confluence. The buffer, plasmid and transfection reagent were added in proportion to the working solution according to the instructions of the jetPRIME Transfection Kit (23Y2307M8; Polyplus, France), and after 4 h, the medium was replaced with fresh antibiotic-free medium and incubated for 24 h. The transfection efficiency was observed by fluorescence microscopy and examined by flow cytometry for further analysis.

### Neutralization of PINK1 in BMSCs

BMSCs were inoculated in a 12-well plate at a concentration of 0.5 × 10^4^ cells/ml. When the cells reached 60–70% confluence, the medium was replaced with fresh medium, and 10 µl of anti-PINK1 (ab23707; 1:1000; Abcam Cambridge, MA, UK) was added. Subsequently, the culture was continued for 24 h to neutralize PINK1 in BMSCs.

### Establishment of the animal kidney IRI model

The mice were randomly divided into five groups: (1) sham operation control (sham) group (*n* = 6); (2) renal ischaemia/reperfusion injury (IRI) group (*n* = 6); (3) untreated BMSC therapy (BMSCs) group (*n* = 6); (4) PINK1-overexpressing BMSC (OE PINK1) group (*n* = 6); and (5) BMSCs with neutralized PINK1 (Anti-PINK1) group (*n* = 6).

In the sham group, the mice were anaesthetized with 2% pentobarbital (40 mg/kg) and fixed on a constant temperature plate at 37 °C. A midline abdominal incision was performed, and the skin, subcutaneous tissue and peritoneum were separated layer by layer. Subsequently, the abdomen was covered with gauze soaked in normal saline for 30 min and then sutured. In the IRI group, the procedure was the same as that in the control group. After opening the abdominal cavity, the left renal pedicle was located and then quickly clipped with a non-invasive artery clamp, followed by timing. Then, the right renal pedicle clip was located, followed by timing. The arterial clip was removed after 30 min to allow the reperfusion of blood flow, and the abdomen was subsequently sutured. In the BMSC group, the IRI-AKI model was established in the same way as in the IRI group, and 0.1 ml of a BMSC cell suspension (1 × 10^7^ cells/ml) was infused through the caudal vein. In the OE PINK1 group, the procedure was the same as that in the BMSC group. PINK1-overexpressing BMSCs (0.1 ml) (1 × 10^7^ cells/ml) were infused through the caudal vein. In the anti-PINK1 group, the procedure was the same as that in the BMSC group. PINK1-neutralized BMSCs (0.1 ml) (1 × 10^7^ cells/ml) were infused through the caudal vein.

### Renal function analysis

After the IRI-AKI mouse model was established successfully, the state of the mice in each group was observed every 24 h. After 72 h, blood was collected from the eyes of the mice in each group and numbered. After being placed at room temperature for 30 min to present obvious stratification, the blood was centrifuged at 3500 r/min for 5 min. The levels of serum creatinine (SCR) and blood urea nitrogen (BUN) were measured on an automatic biochemical analyser (Hitachi 7180, Tokyo, Japan).

### Histopathology

Kidney samples were collected and fixed with formalin. The kidney tissues were subjected to paraffin embedding, and continuous sectioning was performed at a thickness of 4 μm, followed by routine haematoxylin–eosin (HE) staining. An optical microscope (Nikon, Tokyo, Japan) was used for observation. The stained tissue sections were scored with a semiquantitative scale, and a double-blind method was used to assess the degree of tubular injury. Renal tubular injury was characterized by brush border loss r, dilatation and/or atrophy of the renal tubules, degeneration of the renal tubules, the formation and vacuolation of renal tubules, inflammatory cell infiltration or oedema, with scores ranging from 0 to 4. The higher the score, the more serious the injury, which was specifically measured as follows: 0 = normal kidney, 1 = mild injury (the degree of cortex or medulla involvement < 5%), 2 = mild to moderate injury (the degree of cortex or medulla involvement 5–25%), 3 = moderate injury (the degree of cortex or medulla involvement 25–75%), and 4 = severe injury (the degree of cortex or medulla involvement > 75%).

### Immunohistochemistry

The paraffin-embedded tissue slices (thickness 3–4 μm) were dewaxed, rehydrated by continuous immersion in ethanol and sealed with a peroxidase sealing solution for 5 min. The primary antibodies (anti-LC3-B, ab48394; anti-mTOR, ab134903; anti-CD3, ab135372; anti-CD14, ab221678; anti-CD20, ab64088; anti-CD68, ab125212; 1:250, Abcam) were added and incubated with the sections, and the sections were stained with biotin-linked secondary antibodies before being treated with streptavidin (K3461, DACCO Cytomation). Subsequently, the slides were treated with an AEC solution at room temperature for 10 min, and images were taken with a fluorescence microscope (Nikon, Tokyo, Japan).

### Terminal deoxynucleotidyl transferase dUTP nick-end labelling (TUNEL) staining

The TUNEL reaction mixture was prepared with the In Situ Cell Death Test Kit (Roche, 11,684,817,910; Sigma, USA) according to the instructions and placed in an ice box for later use. After routine dewaxing and rehydration, the slides were was placed into 200 ml of citrate buffer with 0.1 M (pH = 6.0) and irradiated in a 350-W microwave for 5 min. Subsequently, the slides were washed twice with PBS for 5 min each time. After the samples were dried, 50 μl of TUNEL reaction mixture was added. The slides were incubated in a constant temperature incubator (Shanghai Sumsung Laboratory Instrument Co., Ltd, China) for 60 min in a humid and dark environment at 37 °C. After being washed with PBS 3 times, the slides were sealed with Antifading medium with DAPI (ZLI-9557, ZSGB-BIO, Beijing, China) and analysed under a fluorescence microscope. Apoptosis in mouse kidney tissues was observed under a fluorescence microscope with a magnification of 400×. The number of TUNEL-positive renal tissue cells in at least 10 light fields in each section of 3 different kidneys in each group was quantitatively calculated. The number of TUNEL-positive renal tissue cells in each square millimetre of kidney tissue was calculated.

### Western blotting

Small amounts of tissue blocks were placed in 2 ml EP tubes, and clean steel beads were added. Then, 300 µl of RIPA buffer containing PMSF (R0020-100; Solarbio, Beijing, China) was added to each tube and homogenized in an automatic homogenizer. After homogenization, the EP tube was placed on ice for 30 min. The lysate was transferred to a 1.5-ml centrifuge tube with a pipette and then centrifuged at 12000 rpm for 5 min at 4 °C, and the supernatant was placed into a 0.5 ml centrifuge tube and stored at − 20 °C. The protein sample was diluted appropriately. The protein concentration was quantified using a BCA protein quantification kit (cx00098; Beyotime, Shanghai, China). The extracted protein was mixed with 5 × protein loading buffer and boiled in a water bath for 10 min until the sample was clear. After denaturation, the samples were cooled to room temperature and then stored at − 20 °C. The prefabricated glue was fixed to the electrophoresis tank, and the electrophoresis solution was poured into the reservoir. The prepared protein samples and MAKER were added to the upper sample wells with a microsampler, and the total protein amount of each sample was 40 μg. Then, the proteins were transferred to PVDF membranes. The PVDF film was soaked in TBST (blocking solution) containing 5% skim milk powder for 2 h at room temperature. The corresponding primary antibody was diluted with blocking solution, and the PVDF membrane was immersed in the primary antibody incubation solution and incubated overnight at 4 °C. The PVDF membranes were adequately washed five times with TBST. The corresponding HRP-labelled secondary antibody was diluted with TBST, and the PVDF membrane was immersed in the secondary antibody incubation solution at room temperature on a shaker for 2 h. The PVDF membranes were adequately washed five times. The enhancement solution in the ECL reagent was mixed with the stable peroxidase solution in a 1:1 ratio, and the working solution was added dropwise onto the PVDF membrane. The reaction lasted for a few minutes after the fluorescent band was visualized. After exposing the X-ray film, the developer liquid was added successively, and then the film was developed.

### Establishment of a BMSC model targeting injured kidney tissues

The left kidney ischaemia–reperfusion model was established as described previously. The RFP-BMSC cell suspension (0.1 ml, 1 × 10^7^ cells/ml) was infused into the caudal vein. The mice were subjected to humane euthanasia after 24 h, and fresh bilateral kidney tissues were collected and frozen to prepare sections. The homing of BMSCs to bilateral tissues was observed with a fluorescence microscope.

### Establishment of the hypoxia-reoxygenation cell model

After the passaged C57BL/6 mouse BMSCs were fused with RTECs to approximately 50%, serum starvation was continued for 24 h. Subsequently, the cell samples were washed with PBS twice, immersed in mineral oil (M5310; Sigma–Aldrich, USA) at 37 °C for 60 min, washed with a large amount of PBS many times, and cultured in complete culture medium for 24 h [16].

### Isolation of peripheral blood mononuclear cells (PBMCs)

Mouse peripheral blood was collected and diluted with an equal volume of saline, centrifuge tubes were prepared and 2 ml of lymphocyte isolates was added. The diluted blood was mixed well, and 2 ml of the diluted blood was gently injected into a centrifuge tube containing 2 ml of the lymphocyte separator along the test tube wall so that the diluted blood was in the upper layer of the lymphocyte separator. Two centrifuge tubes were placed in a centrifuge and centrifuged at 2000 rpm for 20 min. The white membrane layers in the two tubes were absorbed separately and placed in a new centrifuge tube. PBS was added to the white membrane layer cell suspension, mixed and centrifuged at 1500 rpm for 5 min, and the supernatant was discarded. The suspension was mixed with appropriate amounts of PBS according to the amount of cell precipitation and set aside.

### Co-culture of cells

Normal BMSCs, PINK1-overexpressing BMSCs or PINK-1-neutralized BMSCs were co-cultured with hypoxia-reoxygenation C57BL/6 mouse RTECs under conventional conditions (1:10).Normal BMSCs, PINK1-overexpressing BMSCs or PINK-1-neutralized BMSCs were co-cultured with normal C57BL/6 mouse RTECs under conventional conditions (1:10). After the cells reached 60%–80% confluence, they were treated with hypoxia-reoxygenation conditions.

Normal BMSCs, PINK1-overexpressing BMSCs or PINK-1-neutralized BMSCs were co-cultured with PBMCs isolated from normal mice (1:1). Normal BMSCs, PINK1-overexpressing BMSCs or PINK-1-neutralized BMSCs were cultured under hypoxic reoxygenation conditions and then co-cultured with PBMCs isolated from normal mice (1:1). Normal BMSCs, PINK1-overexpressing BMSCs or PINK-1-neutralized BMSCs were co-cultured with PBMCs isolated from mice with bilateral renal ischaemia/reperfusion (1:1). For the next experiment, after 24 h of incubation.

### Apoptosis analysis by flow cytometry (FCM)

Cell preparation was performed with the PE Annexin V Apoptosis Detection Kit I (No. 559763, BD, Franklin Lakes, NJ, USA). The cells were digested with EDTA-free trypsin (BI, Israel). After the cells were collected, they were washed twice with precooled PBS at 4 °C. The concentration of the cells was adjusted to 1 × 10^6^ cells/ml by adding 1 × buffer. A single cell suspension of 1 × 10^5^ cells/tube was added to 5 µl/tube PE Annexin V. After being mixed, these cells were incubated in the dark at room temperature for 15 min. Subsequently, 5 µl/tube 7-AAD and the proper buffer were added to each flow tube. FCM was performed (BD, Franklin Lakes, NJ, USA) within 1 h.

### Cell counting kit‑8 (CCK‑8) assay

A cell suspension of PBMCs (100 µl) was seeded in a 96-well plate. The culture plate was preincubated for 24 h (37 °C, 5% CO_2_). Ten microlitres of CCK solution was added to each well. The culture plate was incubated for 3 h. The absorbance at 450 nm was measured by a microplate reader.

### Enzyme-linked immunosorbent assay (ELISA)

The levels of interleukin-10 (IL-10) and tumour necrosis factor-α (TNF-α) in serum and cell supernatants were examined by ELISA (ELISA kit: EMC102a.96, EMC005.96; Neobioscience, Tianjin, China). The experimental steps were conducted according to the instructions, and the OD450 was measured immediately after completion (within 3 min). The data were saved.

### Transmission electron microscopy (TEM)

Tissue samples were collected, washed once in PBS and placed in an EP tube. Glutaraldehyde (2.5%) was prepared, and the cells were fixed in phosphate buffer for 2 h. The beads were rinsed with 0.1 M phosphoric acid three times. The cells were fixed with 1% osmium acid for 2–3 h and rinsed with 0.1 M phospho rinse three times. In a 4 °C refrigerator, 50%, 70%, and 90% ethanol were successively added and incubated for 15 min, 90% ethanol 90% acetone (1:1) was added for 15 min, and 90% acetone was added for 15 min. The samples were then soaked in 100% acetone three times for 15 min at room temperature. Pure acetone + embedded solution (2:1) was added and incubated in turn for 4 h at room temperature, pure acetone + embedded solution (1:2) was added and incubated overnight at room temperature, and pure embedded liquid was added and incubated at 37 °C for 3 h. The samples were successively incubated overnight at 37 °C, 12 h at 45 °C, and 24 h at 60 °C. Ultrathin sections were prepared at a thickness of 50 nm, double stained with 3% uranyl acetate-lead citrate and visualized by TEM. Five copper nets were observed in each group, and three images were randomly collected in each group.

### Statistical analysis

All statistical analyses were conducted with SPSS 23.0 software. According to the measurement data with normal distribution, the t test or one-way ANOVA was used to analyse and compare the means between groups, and the results are expressed as the mean ± standard deviation. Independent sample t test was used for comparisons between the two groups; one-way ANOVA was used to analyse the differences among multiple groups; the LSD test was used for pairwise comparisons between groups; Dunnett’s T3 test was used for the comparison between groups with a heterogeneity of variance; and the Kruskal–Wallis H test was used to compare nonnormal distribution data and ranked data. P < 0.05 was considered to be statistically significant.

## Results

### PINK1 participated in the protective effect of BMSCs on kidney tissues in IRI-AKI

#### PINK1 was related to the repair of kidney tissues mediated by BMSCs in IRI-AKI mice

**Fig. 1:**
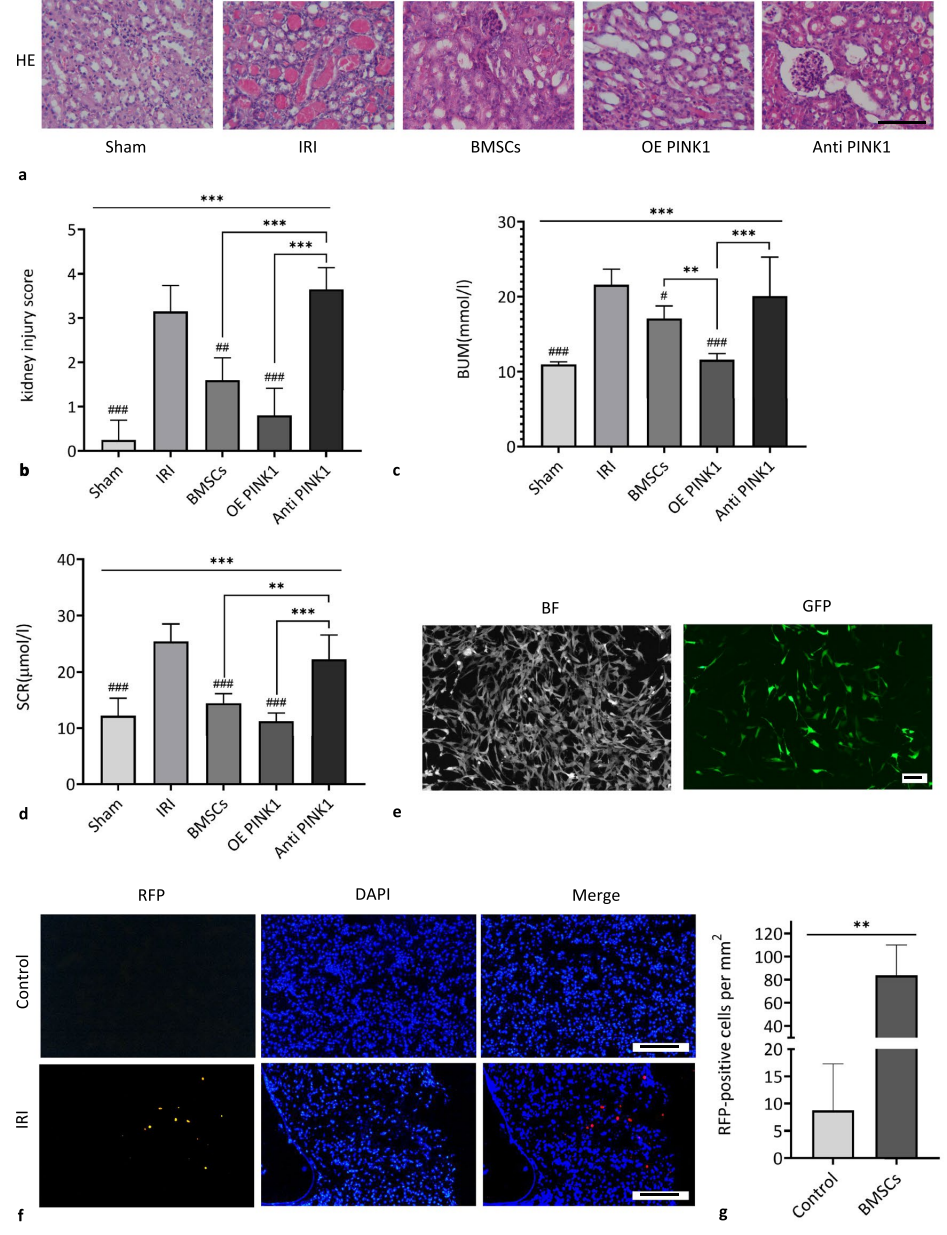
PINK1 enhances BMSC-mediated repair of kidney tissues in IRI-AKI mice. **a** Representative image of HE staining, × 200, scale: 100 μm. **b** Pathological score of renal tubular injury (*n* = 6). **c** The levels of BUN in blood samples (*n* = 6). **d** The levels of SCR in blood samples (*n* = 6). **e** BMSCs were successfully transfected with GFP-PINK1, × 100, BAR: 100 μm. **f** RFP-BMSCs in kidney tissues were observed with a fluorescence microscope. × 200, BAR: 100 μm. **g** Quantitative analysis of RFP-BMSCs in injured tissues. SEM, ###*p* < 0.001, ##*p* < 0.01 and #p < 0.05, compared with the IRI group; ***p < 0.001, **p < 0.01 and *p < 0.05, compared with among the groups

First, pathological changes in kidney tissues in the IRI-AKI mouse model were observed. Histological examination of HE staining showed that the kidney structure in the sham group was normal under physiological conditions, while renal tubular injury was obvious in the IRI group. Among the three BMSC intervention groups (the BMSC group, OE PINK1 group and Anti-PINK1 group), the OE PINK1 group had the most moderate kidney injury, followed by the IRI group, and the Anti-PINK1 group had the most serious kidney injury (Fig. 1a). Based on the kidney injury score, the degree of renal tubular injury in the OE PINK1 group (0.80 ± 0.62) was significantly lower than that in the IRI group (3.15 ± 0.59) (*P* < 0.001) and the Anti-PINK1 group (3.65 ± 0.49) (*P* < 0.001), and there was no significant difference between the OE PINK1 group and the sham group (0.25 ± 0.44) (*P* = 0.167). The level of injury in the OE PINK1 group was lower than the BMSC group (1.60 ± 0.50) (*P* = 0.056), but there was no significant difference (Fig. 1b). Consistent with the histological results, the OE PINK1 group had lower concentrations of SCR (11.22 ± 1.48 μmol/L) and BUN (11.57 ± 0.83 mmol/L) than the other groups, and there was a significant difference between the OE PINK1 group and the IRI group (SCR: 25.45 ± 3.10 μmol/L, BUN: 21.61 ± 2.08 mmol/L) (*P* < 0.001; P < 0.001). There was also a significant difference in the expression level of BUN between the OE PINK1 group and the BMSC group (17.10 ± 1.69 mmol/L) (*P* = 0.01), while the concentrations of SCR and BUN in the IRI group and the Anti-PINK1 group (SCR: 22.24 ± 4.36 μmol/L, BUN: 20.08 ± 5.21 mmol/L) were significantly increased (Fig. 1c and d). These results indicated that renal dysfunction occurred in mice with IRI and that BMSCs could improve renal function and repair injured kidney tissues. In addition, PINK1 overexpression could enhance BMSC-mediated repair, and neutralizing PINK1 could impair BMSC-mediated repair.

In addition, the characteristics of BMSC targeting to injured kidney tissues were verified by infusing BMSCs with fluorescent labels into the mouse caudal vein in the unilateral kidney injury model and observing the number of fluorescent cells in injured and normal kidneys in the same mouse, as shown in Fig. 1f. The number of fluorescent cells on the injured side was relatively higher (83.75 ± 26.26/mm^2^), and there was a significant difference compared with that on the control side (8.75 ± 8.5/mm^2^, t = 5.432, *P* = 0.002), as shown in Fig. 1g. These results indicated that BMSCs could target injured tissues.

#### PINK1 was related to BMSC-mediated inhibition of apoptosis in the kidney tissues of IRI-AKI mice

**Fig. 2:**
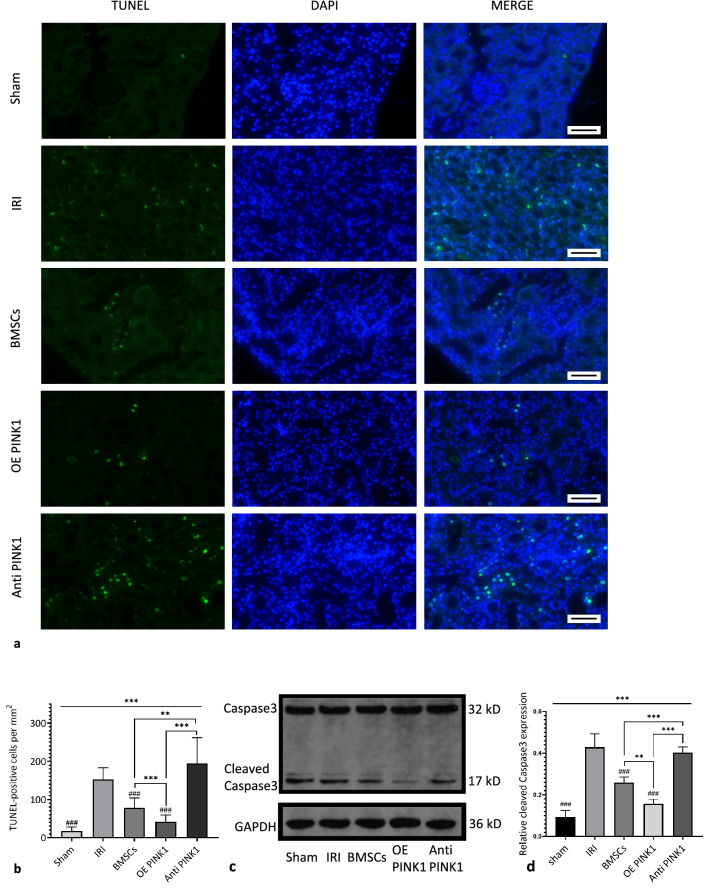
PINK1 enhances the inhibitory effect of BMSCs on apoptosis in the kidney tissues of IRI-AKI mice. **a** Representative image of TUNEL staining in kidney tissues. **b** The number of TUNEL-positive cells in each square millimetre of kidney tissue slices, *n* = 30, × 400, Bar: 50 μm. **c** The relative expression levels of cleaved caspase-3 and the internal reference protein GAPDH in renal tissues were examined by western blotting. **d** Quantitative analysis of the relative expression levels of cleaved Caspase3 in renal tissues in each group. SEM, ###*p* < 0.001, ##*p* < 0.01 and #*p* < 0.05, compared with the IRI group; ***p < 0.001, **p < 0.01 and *p < 0.05, compared with among the groups

TdT-mediated dUTP nick-end labelling (TUNEL) was used to examine apoptosis in kidney tissues, and the effect of PINK1 overexpression and neutralization on apoptosis in kidney tissues was observed. As shown in Fig. 2a and b, the number of TUNEL-positive cells in kidney tissue in the OE PINK1 group was significantly decreased (41.5 ± 17.93/mm^2^), while the IRI group (156.87 ± 26.38/mm^2^) and the Anti-PINK1 group (194.22 ± 67.79/mm^2^) had increased numbers of these cells. These results indicated that the number of apoptotic cells in the BMSC group (86.32 ± 18.44/mm^2^) was significantly lower than that in the IRI group (*P* < 0.001), and the number of TUNEL-positive cells in kidney tissues in the OE PINK1 group and the Anti-PINK1 group was significantly different from that in the BMSC group (*P* < 0.001, *P* < 0.001) (Fig. 2b). These results suggested that BMSCs could play a protective role in the kidney tissues of IRI-AKI mice and reduce apoptosis induced by IRI. In addition, PINK1 overexpression enhanced the effect of BMSCs on apoptosis in injured tissues, while PINK1 neutralization weakened the protective effect of BMSCs.

Second, the relative expression level of the apoptotic protein Caspase3 in different renal tissues was determined by western blotting, and the relative expression level of activated Caspase3 (cleaved Caspase3) was mainly observed. The relative expression level of cleaved Caspase3 in the BMSC group (0.26 ± 0.03) was decreased compared with that in the IRI group (0.43 ± 0.06). The expression level in the OE PINK1 group (0.16 ± 0.02) was lower than that in the BMSC group (*P* = 0.008) and significantly lower than that in the IRI group (*P* < 0.001) and the Anti-PINK1 group (0.40 ± 0.03, *P* < 0.001), and there was no significant difference from that in the sham group (0.09 ± 0.03, *P* = 0.07) (Fig. 2c, d). The expression level of apoptotic proteins was consistent with the trend in apoptosis in tissues as measured by the TUNEL assay, further verifying that PINK1 overexpression could enhance the effect of BMSCs and reduce apoptosis in damaged tissues.

#### PINK1 participated in BMSC-mediated alleviation of the inflammatory reaction in kidney tissues in IRI-AKI mice

**Fig. 3:**
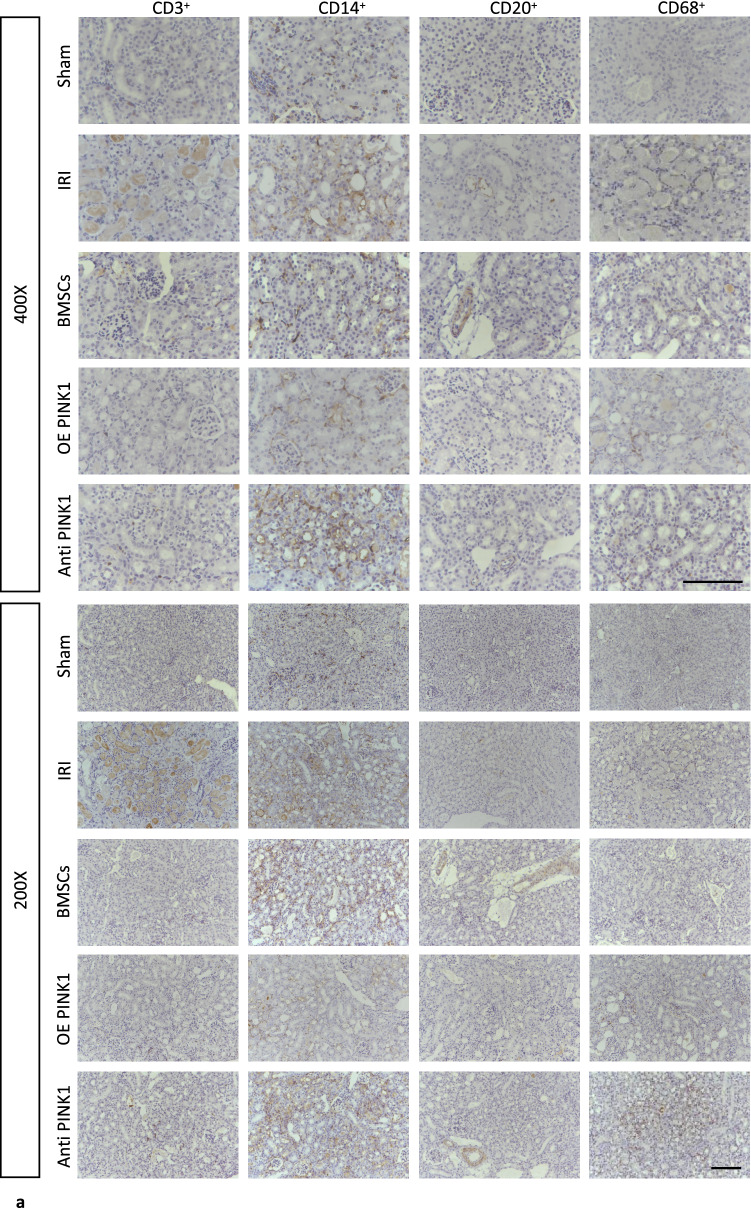

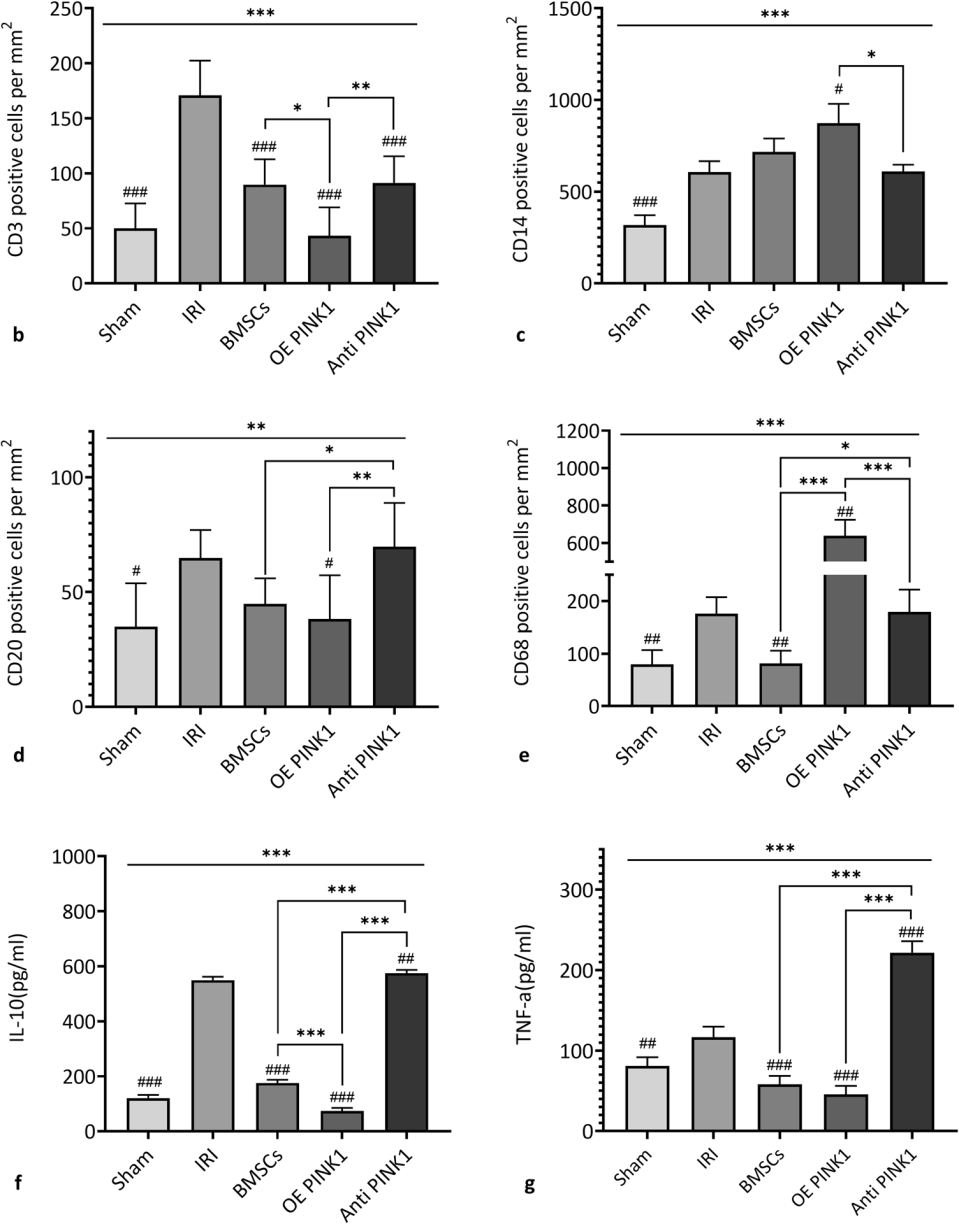
PINK1 enhances the anti-inflammatory effect of BMSCs on the kidneys of IRI-AKI mice. **a** Representative image of immunohistochemical staining. × 400 and × 200, BAR: 100 μm. **b** CD3-positive cell counts (*n* = 6). **c** CD14-positive cell counts (*n* = 6). **d** CD20-positive cell counts (n = 6). **e** CD68-positive cell counts (*n* = 6). **f** Relative changes in the expression of IL-10 in peripheral blood (*n* = 6). **g** Relative changes in the expression of TNF-α in peripheral blood (*n* = 6). SEM, ###*p* < 0.001, ##*p* < 0.01 and #*p* < 0.05, compared with the IRI group; ***p < 0.001, **p < 0.01 and *p < 0.05, compared with among the groups

Inflammation plays an important role in the occurrence and progression of IRI-AKI. Therefore, renal inflammation was evaluated by observing the infiltration of renal immune cells (macrophages, monocytes, T cells, and B cells) by immunohistochemical staining, as shown in Fig. 3a. The expression of CD3^+^ in the IRI group (170.98 ± 31.39/mm^2^) was significantly higher than that in the sham group (49.80 ± 22.73/mm^2^) (*P* < 0.001), the BMSC group (89.64 ± 23.03/mm^2^) (*P* < 0.001) and the OE PINK1 group (43.16 ± 25.85/mm^2^) (*P* < 0.001) had relatively lower levels than the IRI group, and the OE PINK1 group had a relatively lower level than the BMSC group (*P* = 0.01) and the Anti-PINK1 group (91.30 ± 24.20/mm^2^) (*P* = 0.008), as shown in Fig. 3b. The expression of CD14^+^ in the IRI group (605.90 ± 59.56/mm^2^) was significantly higher than that in the sham group (317.06 ± 54.55/mm^2^) (*P* < 0.001), the OE PINK1 group (873.16 ± 105.87/mm^2^) had a relatively higher level than the IRI group (*P* = 0.017) and the Anti-PINK1 group (609.22 ± 37.40/mm^2^) (*P* = 0.023), and the OE PINK1 group had a higher level than the BMSC group (715.46 ± 73.87/mm^2^), but the difference was not statistically significant (*P* = 0.192), as shown in Fig. 3c. There were similar changes in the expression levels of CD20^+^ and CD3^+^, and the differences were not significant. Compared with the IRI group (64.74 ± 12.31/mm^2^), the sham group (34.86 ± 18.93/mm^2^) (*P* = 0.01) and the OE PINK1 group (38.18 ± 19.11/mm^2^) (*P* = 0.019) had significantly lower levels of expression, the Anti-PINK1 group (69.72 ± 19.11/mm^2^) had a slightly higher level than the BMSC group (44.82 ± 11.14/mm^2^) (*P* = 0.027) and the OE PINK1 group (*P* = 0.007), and the OE PINK1 group had a lower level than the BMSC group, but the difference was not statistically significant (P = 0.532), as shown in Fig. 3d. The expression of CD68^+^ in the IRI group (175.96 ± 31.28/mm^2^) was significantly higher than that in the sham group (79.68 ± 27.28/mm^2^) (*P* = 0.007) and the BMSC group (81.34 ± 24.48/mm^2^) (*P* = 0.007) and was significantly lower than that in the OE PINK1 group (640.76 ± 86.73/mm^2^) (*P* < 0.001); the OE PINK1 group had a relatively higher level than the BMSC group (*P* < 0.001) and the Anti-PINK1 group (179.28 ± 42.56/mm^2^) (*P* < 0.001), as shown in Fig. 3e. These results demonstrated that IRI could induce the infiltration of lymphocytes, macrophages, monocytes and B cells in injured tissues, while BMSC infusion could reduce the infiltration of lymphocytes, B cells and macrophages and increase the infiltration of monocytes. PINK1 enhanced the effect of BMSCs on decreasing the infiltration of lymphocytes and B cells and increasing the infiltration of macrophages and monocytes. PINK1 neutralization inhibited this effect.

Moreover, the serum levels of the inflammatory cytokines interleukin-10 (IL-10) and tumour necrosis factor-α (TNF-α) in the peripheral blood of IRI-AKI mice were analysed by ELISA. The results showed that compared with the sham group(IL-10: 120.27 ± 11.81 pg/ml; TNF-α: 80.90 ± 10.90 pg/ml), the IRI group had an increased levels of IL-10 (549.42 ± 12.58 pg/ml, *P* < 0.001) and TNF-α (116.69 ± 13.06 pg/ml, *P* = 0.01); the BMSCs group (IL-10: 175.54 ± 11.69 pg/ml, *P* < 0.001; TNF-α: 45.70 ± 10.60 pg/ml, *P* < 0.001) had significantly decreased levels compared with the IRI group. The OE PINK1 group (73.32 ± 11.72 pg/ml, *P* < 0.001) had a lower level of IL-10 than the BMSCs group, but there was no significant difference in the expression level of TNF-α (58.14 ± 10.60 pg/ml, *P* = 0.176) between the two groups. The Anti-PINK1 group (IL-10: 575.38 ± 11.18 pg/ml, *P* < 0.001; TNF-α: 221.70 ± 14.30 pg/ml, *P* < 0.001) had higher levels of both inflammatory cytokines than the BMSCs group, as shown in Fig. 3f and g. These results indicated that BMSCs could alleviate the inflammatory reaction in kidney tissues in IRI-AKI mice, and PINK1 could further enhance the anti-inflammatory effect of BMSCs. These results suggested that the anti-inflammatory effect of BMSCs on IRI-AKI were enhanced by PINK1 and may be related to macrophages and monocytes.

### Stress response of BMSCs under hypoxia-reoxygenation conditions

#### PINK1 reduced BMSC apoptosis under hypoxia-reoxygenation conditions

**Fig. 4:**
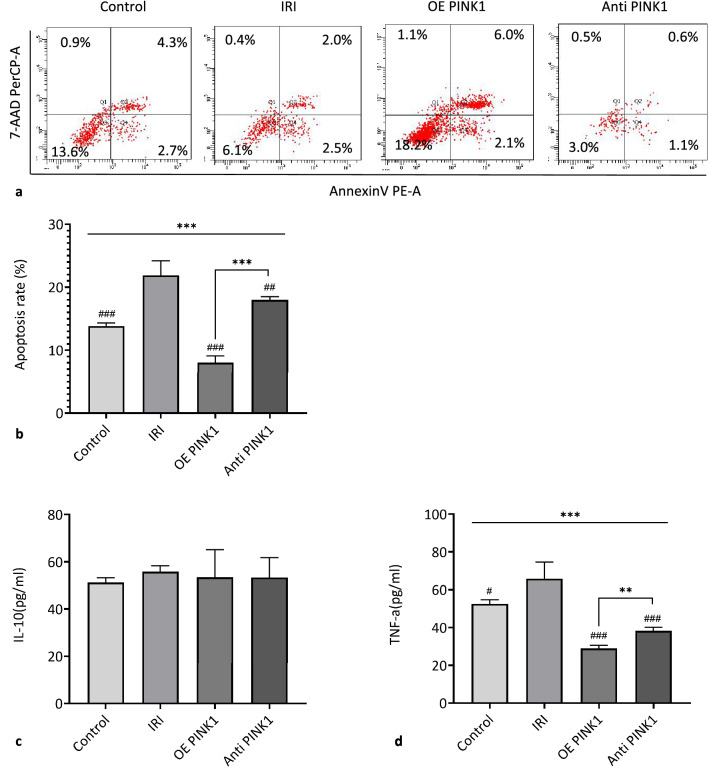
PINK1 mitigates the stress response of BMSCs under hypoxia-reoxygenation conditions. **a** Representative image of BMSC apoptosis as examined by flow cytometry. **b** Quantitative analysis of apoptosis (*n* = 3). **c** Relative changes in the expression level of IL-10 in the cell supernatant (*n* = 3). **d** Relative changes in the expression level of TNF-α in the cell supernatant (*n* = 3). SEM, ###*p* < 0.001, ##*p* < 0.01 and #*p* < 0.05, compared with the IRI group; ***p < 0.001, **p < 0.01 and *p < 0.05, compared with among the groups

An in vitro hypoxia-reoxygenation model was established to simulate the in vivo IRI environment. FCM was used to evaluate apoptosis in BMSCs, PINK1-overexpressing BMSCs and PINK1-neutralized BMSCs under hypoxia-reoxygenation conditions, as shown in Fig. 4a. Compared with the control group (13.50 ± 0.58), the IRI group (21.50 ± 2.08, *P* < 0.001) had a significantly increased apoptosis rate, the OE PINK1 group (8.05 ± 1.07, *P* < 0.001) had a significantly decreased apoptosis rate compared with the IRI group, and the Anti-PINK1 group (18.00 ± 0.53, *P* = 0.002) had a decreased apoptosis rate compared with the IRI group, as shown in Fig. 4b. These results indicated that BMSC apoptosis increased under hypoxia stress and that PINK1 reduced BMSC apoptosis under hypoxia-reoxygenation conditions.

#### *PINK1 was reduced during the inflammatory response of BMSCs under hypoxia-reoxygenation conditions*.

In this study, an in vitro hypoxia-reoxygenation model was established to simulate the in vivo IRI environment. FCM was used to evaluate the expression level of inflammatory factors in BMSCs, PINK1-overexpressing BMSCs and PINK1-neutralized BMSCs under hypoxia-reoxygenation conditions, and there was no significant change in the expression level of IL-10 (*P* = 0.834). Compared with those in the control group (52.49 ± 2.22 pg/ml), the IRI group (65.94 ± 8.71 pg/ml, *P* = 0.027) had significantly increased TNF-α levels, the OE PINK1 group (28.90 ± 1.76 pg/ml, *P* = 0.030) had significantly decreased levels, and the Anti-PINK1 group (38.22 ± 1.98 pg/ml, *P* < 0.01) had significantly decreased levels, as shown in Fig. 4c and d. These results indicated that BMSCs had increased levels proinflammatory factors under hypoxia stress, and PINK1 could reduce the release of proinflammatory factors under hypoxia-reoxygenation conditions.

### PINK1 participated in the stress response of BMSCs to relieve RTECs under hypoxia-reoxygenation conditions

#### PINK1 participated in BMSC-mediated inhibition of apoptosis in RTECs under hypoxia-reoxygenation conditions

**Fig. 5:**
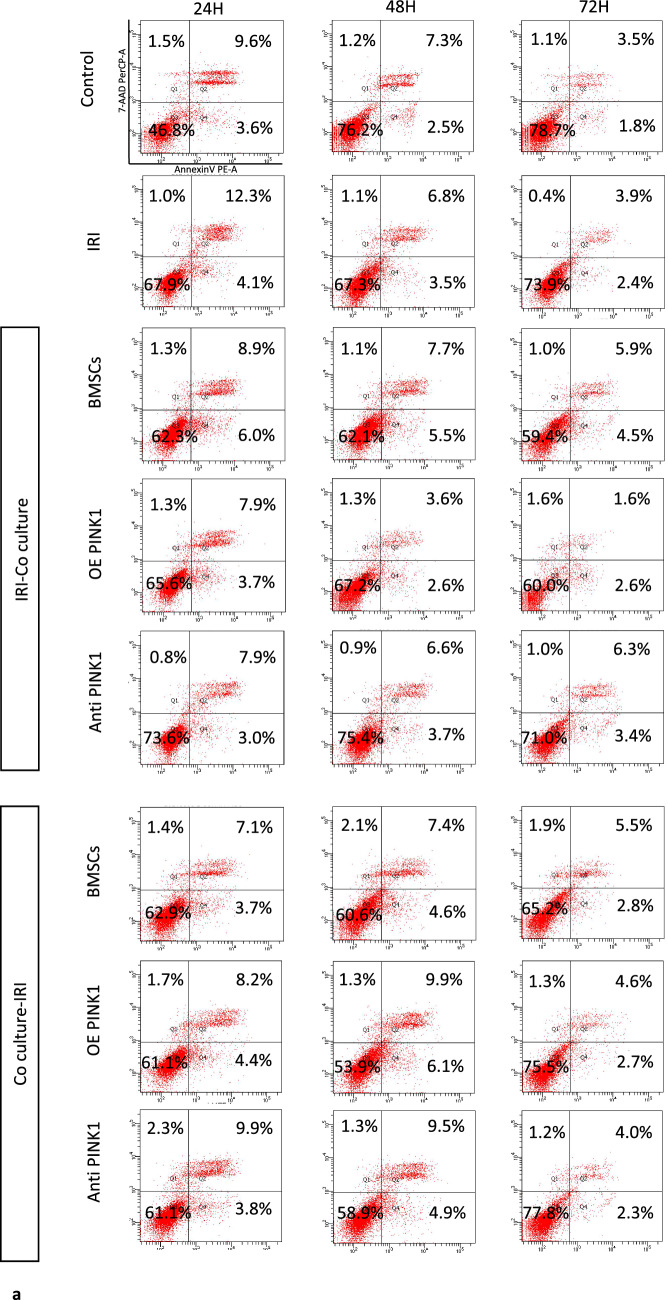

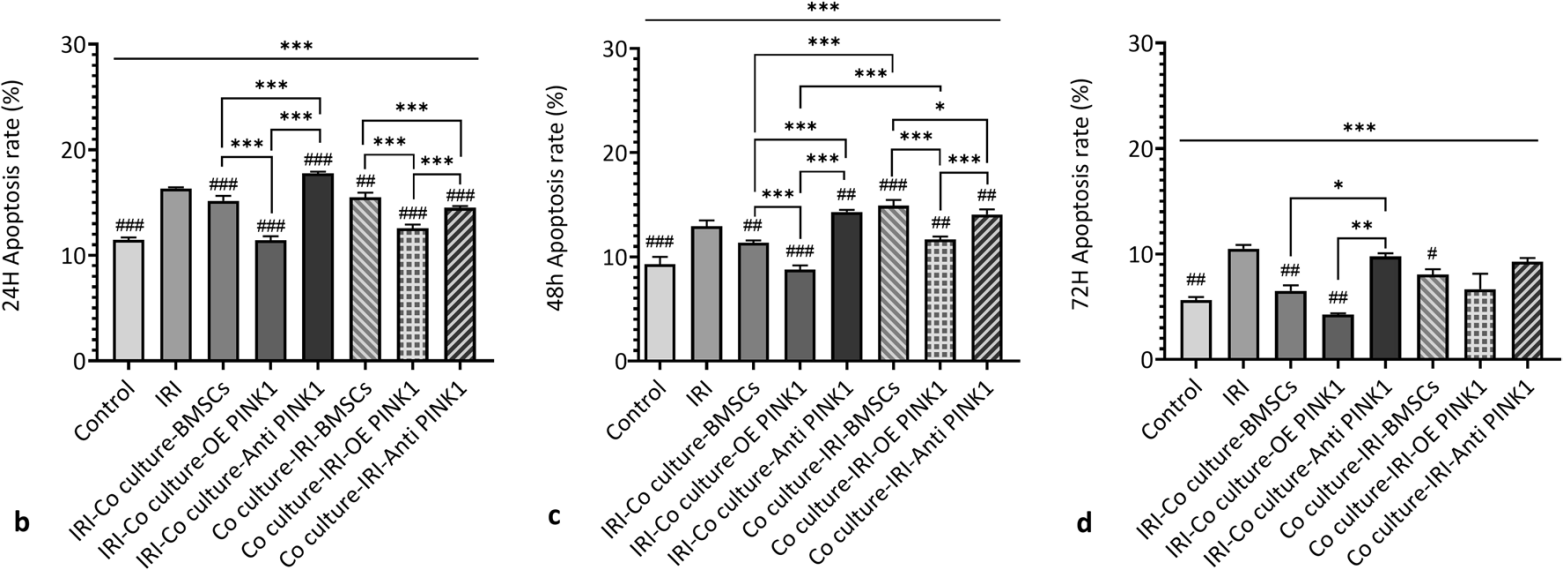
PINK1 enhances the BMSC-mediated reduction in RTEC apoptosis under hypoxia-reoxygenation conditions. **a** Representative image of apoptosis was examined by FCM. **b** Quantitative analysis of the 24-h apoptosis rate. **c** Quantitative analysis of the 48-h apoptosis rate. **d** Quantitative analysis of the 72-h apoptosis rate. SEM, ###*p* < 0.001, ##*p* < 0.01 and #*p* < 0.05, compared with the IRI group; ***p < 0.001, **p < 0.01 and *p < 0.05, compared with among the groups

In this study, an in vitro hypoxia-reoxygenation model was established to simulate the in vivo IRI environment. RTECs in the three BMSC intervention groups (the BMSC group, OE PINK1 group and Anti-PINK1 group) were evaluated before and after hypoxia and reoxygenation. RTECs co-cultured with BMSCs after ischaemia–reperfusion injury were divided into the IRI-co-culture group, while RTECs co-cultured before hypoxia and reoxygenation were divided into the co-culture-IRI group. The effects on RTEC apoptosis were observed after 24 h, 48 h and 72 h, as shown in Fig. 5a. Our study showed that the apoptosis rate decreased gradually with time, as shown in Fig. 5b–d. At 24 h and 48 h, the apoptosis rate in each group was significantly different from that in the IRI group (24 h: 16.33 ± 0.12, 48 h: 12.93 ± 0.55). Regardless of the order of co-culture, the apoptosis rate in the OE PINK1 group was significantly lower than that in the BMSC group (*P* < 0.001, *P* < 0.001, *P* < 0.001, *P* < 0.001) (Fig. 5b–c). The apoptosis rate in the IRI-co-culture-BMSC group (11.37 ± 0.21) was significantly lower than that in the co-culture-IRI-BMSC group (14.90 ± 0.56) (*P* < 0.001) at 48 h, and the apoptosis rate in the IRI-co-culture-OE PINK1 group (8.77 ± 0.40) was significantly lower than that in the co-culture-IRI-OE PINK1 group (11.67 ± 0.29) (*P* < 0.001), as shown in Fig. 5c. The results at 48 h showed that BMSCs without hypoxic reoxygenation stimulation may have better antiapoptotic abilities, and these results indicated that PINK1 overexpression could reduce apoptosis induced by hypoxia and reoxygenation and enhance the survival of BMSCs during the stress response.

#### *PINK1 overexpression *in vitro* enhanced the resistance of BMSCs to the inflammatory response of RTECs under hypoxia-reoxygenation conditions, altering the release of inflammatory factors and reducing the proliferation of PBMCs*

**Fig. 6:**
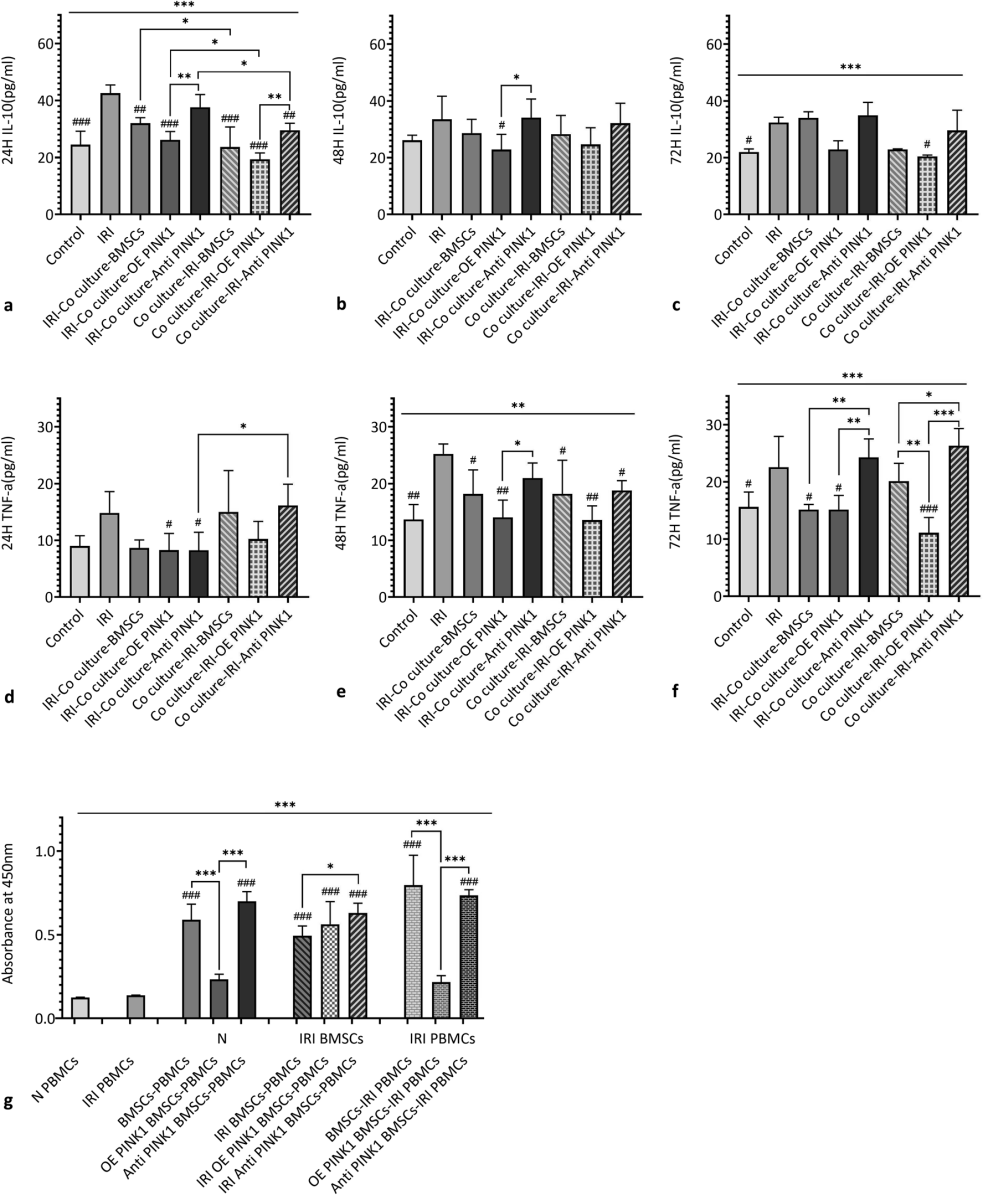
PINK1 enhances the release of inflammatory factors from co-cultured BMSCs and RTECs under hypoxia-reoxygenation conditions. **a**, **b** and **c** The expression level of IL-10 in the co-culture supernatant after 24 h, 48 h and 72 h. **d**, **e** and **f** The expression level of TNF-α in the co-culture supernatant after 24 h, 48 h and 72 h. **g** Absorbance analysis of the proliferation of PBMCs as examined by CCK-8 assays. SEM, ###*p* < 0.001, ##*p* < 0.01 and #*p* < 0.05, compared with the IRI group; ***p < 0.001, **p < 0.01 and *p < 0.05, compared with among the groups

Based on the groupings described above, the effect of inflammatory factor release in the co-culture supernatant was observed at 24 h, 48 h and 72 h, as shown in Fig. 6a–f. There was no significant difference in the release of inflammatory factors after 48 h (*P* = 0.26). The ELISA results revealed that the expression level of IL-10 in the IRI-co-culture group was higher than that in the co-culture group at all three time points, and the change was significant at 24 h (*P* < 0.001). The expression level of IL-10 in the IRI-co-culture-OE PINK1 group (26.16 ± 2.94 pg/ml) was higher than that in the co-culture-IRI-OE PINK1 group (19.35 ± 2.24 pg/ml) at 24 h (*P* = 0.049), and the expression level of IL-10 in the OE PINK1 group was significantly lower than that in the Anti-PINK1 group regardless of the co-culture sequence (*P* = 0.002; *P* = 0.006). While the expression level of TNF-α changed significantly at 72 h (*P* < 0.001), the expression level of TNF-α in the co-culture-IRI-OE PINK1 group (11.11 ± 2.67 pg/ml) was significantly lower than that in the co-culture-IRI-BMSC group (20.13 ± 3.12 pg/ml) (*P* = 0.003) and the co-culture-IRI-Anti-PINK1 group (26.33 ± 2.99 pg/ml) (*P* < 0.001). These results indicated that PINK1 overexpression could reduce the release of inflammatory factors induced by hypoxia and reoxygenation.

Moreover, after PBMCs were isolated from mice, the proliferation of PBMCs was evaluated by CCK-8 assays. Normal BMSCs and hypoxia-reoxygenation BMSCs were co-cultured with PBMCs isolated from normal mice and mice with IRI, respectively. Changes in BMSC proliferation were measured to examine the anti-inflammatory effect of BMSCs in vivo, as shown in Fig. 6g. Compared with the IRI PBMCs, BMSCs (*P* < 0.001) and hypoxia-reoxygenation BMSCs (*P* < 0.001) promoted the proliferation of PBMCs, and PINK1 overexpression significantly reduced the effects of BMSCs on the proliferation of PBMCs and IRI PBMCs (*P* < 0.001; *P* < 0.001). Anti-PINK1-treated BMSCs (*P* < 0.001; *P* < 0.001; *P* < 0.001) promoted the proliferation of PBMCs. These results indicated that there were rapid changes in PBMCs during the response to external oxidative stress, and BMSCs generated a series of effects during the response to oxidative stress, and these effects stimulated the proliferation of PBMCs. PINK1 overexpression reduced the effect of BMSCs on PBMC proliferation.

### PINK1 participated in IRI-AKI by targeting mitophagy

**Fig. 7:**
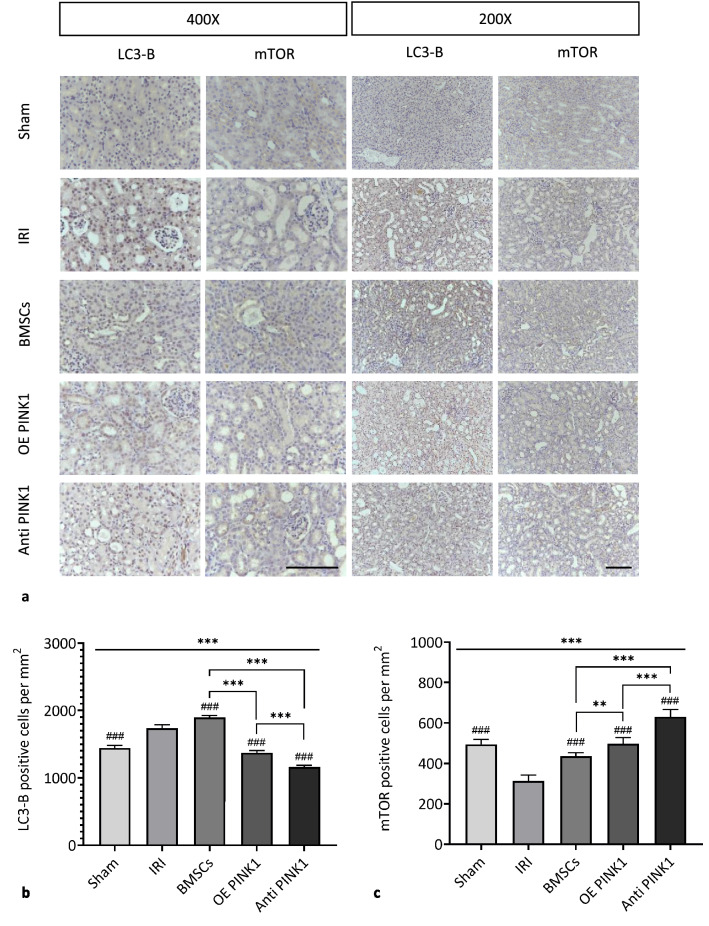

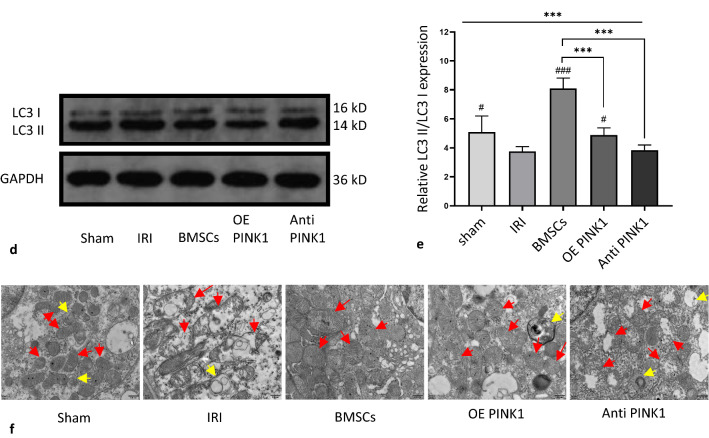
PINK1 participates in IRI-AKI by targeting mitophagy. **a** Representative image of LC3-B and mTOR, as shown by immunohistochemical staining in kidney tissues. × 400 and × 200, BAR: 100 μm. **b** The number of cells that were positive for LC3-B by immunohistochemical staining. **c** The number of cells that were positive for mTOR by immunohistochemical staining. **d** Representative images showing the relative expression levels of LC3II/LC3I and the reference protein GAPDH as examined by western blotting. **e** Quantitative analysis of the relative expression levels of LC3II/LC3I in renal tissues in each group. **f** Representative TEM images of mitochondrial morphology in each group of mice, BAR: 500 nm, Red arrow: mitochondrial damage; Yellow arrow: autophagosome. SEM, ###*p* < 0.001, ##*p* < 0.01 and #*p* < 0.05, compared with the IRI group; ***p < 0.001, **p < 0.01 and *p < 0.05, compared with among the groups

PINK1 regulates mitophagy, and IRI-AKI is closely related to autophagy. The immunohistochemical results shown in Fig. 7a-c indicated that compared with that in the IRI group (LC3-B: 1738.02 ± 50.35/mm^2^; mTOR: 312.08 ± 30.27/mm^2^), the expression level of light chain 3 (LC3)-B in the sham group (1442.54 ± 37.76/mm^2^) was decreased (*P* < 0.001), and the expression level of mammalian target of rapamycin (mTOR) (494.68 ± 23.91/mm^2^) was increased (*P* < 0.001). The expression level of LC3-B in the BMSC group (1900.70 ± 24.20/mm^2^) was decreased (*P* < 0.001), and the expression level of mTOR (434.92 ± 17.21/mm^2^) was increased (*P* < 0.001). Compared with that in the BMSC group, the expression level of LC3-B in the OE PINK1 group (1371.16 ± 33.92/mm^2^) was increased (*P* < 0.001), and the expression level of mTOR (496.34 ± 31.28/mm^2^) was decreased (*P* < 0.001), which was similar to the results in the Anti-PINK1 group (LC3-B: 1163.66 ± 21.48/mm^2^; mTOR: 630.80 ± 36.18) (*P* < 0.001; *P* < 0.001). These results indicated that the level of LC3-B was inversely correlated with mTOR expression levels and that BMSCs could enhance mitophagy and repair injured tissues by increasing the expression level of LC3-B and decreasing the expression level of mTOR. The expression levels of LC3-B and mTOR in the OE PINK1 group were between those in the BMSC group and the Anti-PINK1 group. Our results showed that PINK1 overexpression could enhance the reparative effect of BMSCs on IRI-AKI renal tissue. Moreover, due to the dual effects of mitophagy, too much and too little mitophagy can cause tissue damage, and the mechanism by which PINK1 overexpression enhances the effect of BMSCs on renal tissue repair may involve maintaining mitophagy homeostasis by regulating autophagy levels.

The relative expression level of the autophagy-related protein LC3II/LC3I in each group of tissues was examined by western blotting, as shown in Fig. 7d and e. The relative expression level of LC3II/LC3I was decreased in the IRI group (3.75 ± 0.33) compared to the sham group (5.09 ± 1.11) (*P* = 0.034). Compared with that in the IRI group, the expression level in the BMSC group (8.10 ± 0.71) was significantly increased (*P* < 0.001). Compared with that in the BMSC group, the relative expression level of LC3II/LC3I in the OE PINK1 group (34.89 ± 0.49) was significantly reduced (*P* < 0.001). The expression in the Anti-PINK1 group (3.84 ± 0.36) was lower than that in the OE PINK1 group (*P* < 0.001). The expression levels of the OE PINK1 group were between those of the BMSC and Anti-PINK1 groups, which was consistent with histochemistry results. Furthermore, PINK1 overexpression enhanced the effect of BMSCs on renal tissue repair by regulating mitochondrial autophagy and maintaining mitochondrial homeostasis, thus protecting renal function.

Next, the mitochondria and the ultrastructure of autophagy in renal tissue in each group of mice were observed by TEM, as shown in Fig. 7f. Mitochondria structure and distribution in sham group was normal, and mitochondrial damage was less severe with blurred cristae (red arrow). In the IRI group, the cytoplasm showed focal dissolution (blank area), mitochondrial swelling was obvious, and there was severe cristae fracture or loss. In the BMSC group, the damage degree was mild, and some crest breaks disappeared. Compared with the other groups, the OE PINK1 group had clear mitochondrial structures, mild mitochondrial damage and blurred cristae. The Anti-PINK1 group had severe mitochondrial damage, with cristae breaks and loss, and blank areas appeared. These results indicate that PINK1 can enhance BMSC-mediated repair of IRI-AKI kidney tissue by targeting mitochondrial autophagy.

## Discussion

Recent studies have reported that renal IRI-AKI may be related to disordered cellular immune regulation and that IRI-AKI damages renal tissue. In addition, inflammatory cytokines and immune cell infiltration play pivotal roles in kidney diseases. The extensive and complex molecular cROS reactions between RTECs and immune cells, interstitial cells and endothelial cells can regulate kidney recovery. As revealed in a tissue biopsy, T cells or macrophages in renal parenchyma are rare under normal conditions, while inflammatory infiltration of activated T cells and macrophages during IRI-AKI is extensively present in ischaemic kidneys. Furthermore, CD68^+^ cells infiltrate around the glomeruli and renal tubules to different degrees [17, 18]. These findings are consistent with the results of the present study. Additionally, some studies report that there is a shift in the macrophage phenotype from M1 to M2 and the transformation of phagocytic function to structural repair, combined with the formation of new capillaries and the regeneration of renal tubules. It is worth noting that BMSCs can modify inflammatory cells under ischaemic and hypoxic conditions to facilitate the transformation of the immunophenotype [19]. In this study, as shown in Fig. 3e, the significant increase in CD68^+^ macrophages in the OE PINK1 group suggests that BMSCs overexpressing PINK1 may be involved in the regulation of macrophage phenotypic transformation. BMSCs present immunosuppressive effects only when they are exposed to a sufficiently high level of proinflammatory cytokines, which can promote tissue repair and inhibit the occurrence of inflammatory reactions [20]. As highlighted in many studies, the immunomodulatory characteristics of BMSCs are closely related to the immune response mediated by T cells, B cells and NK cells. In addition, BMSCs could participate in the immune response by regulating the activation, expansion and transformation of these cells and the expression profile of proteins that play important roles in their immune function [21, 22]. The important role of mitochondria in regulating the dynamic network of innate and acquired immune signalling pathways has been emphasized in recent studies. As a key enzyme that maintains mitochondrial homeostasis, PINK1 participates in the occurrence and development of many diseases, such as IRI [23, 24]. Mitophagy mediated by PINK1 can eliminate damaged mitochondria, maintain microenvironmental homeostasis and inhibit the occurrence of the immune response. According to the results of this study, PINK1 decreases the infiltration of lymphocytes and B cells and increases the infiltration of macrophages and monocytes in response to treatment with BMSCs (Fig. 3). This finding indicates that PINK1 could enhance the ability of BMSCs to inhibit the immune response by regulating the distribution of immune cells in the damaged kidney during IR. Moreover, unlike the expression level of the anti-inflammatory factor IL-10 in the serum of the model animals in this study, it is thought that endogenous IL-10 comes from immune cells in different tissues (Fig. 3; Fig. 4), and the changes in IL-10 expression correlated with altered immune cell distribution (Fig. 3). PINK1 and BMSCs can synergistically regulate and alleviate immune disorders in kidneys with IRI-AKI and enhance the anti-inflammatory and immunosuppressive properties of BMSCs, thus promoting the repair of kidneys with IRI-AKI.

In addition, clinical and basic trials show that BMSCs are effective in the treatment of AKI. BMSCs are recruited to injured tissues and release certain cytokines and growth factors, such as insulin-like, hepatocyte and vascular endothelial growth factors. These cytokines can activate endogenous cell repair programs, which can promote the growth and survival of endothelial cells and tubular epithelial cells, thus promoting the repair and regeneration of injured tissues [25]. However, although BMSCs have therapeutic potential, their survival rate and biological activity are relatively low under inflammatory and oxidative stress conditions at the injured site, which restricts their usage. Some studies have revealed that under IRI-AKI conditions, the increased oxygen-free radicals produced by mitochondrial damage in kidney tissues are not conducive to the survival and differentiation of infused BMSCs. Pretreatment with hypoxia can improve the survival rate of BMSCs and promote mitochondrial quality control by increasing mitophagy, thus increasing the therapeutic potential of stromal cells [26, 27]. Studies have reported that berberine (BBR) activates mitophagy through PINK1/Parkin and reduces the accumulation of ROS to antagonize AKI induced by the nephrotoxicity of cisplatin [28]. Pioglitazone can repair mitochondrial dysfunction induced by uraemia by upregulating PINK1 expression, inhibiting mitochondrial fusion and promoting mitophagy, thus reducing the degree of damage associated with chronic kidney diseases (CKDs) [29]. The deletion of regulator of calcineurin 1 (RCAN1) in RTECs could alleviate the dysfunction caused by tubulointerstitial fibrosis in CKD by regulating mitophagy induced by PINK1/Parkin [30]. Consistent with the results of this study, PINK1, which is a key mitophagy enzyme, can directly regulate mitophagy to improve IRI-AKI. Han et al. [31] showed that melatonin could upregulate PrPC expression and binding to PINK1. Melatonin can promote mitochondrial dynamics and metabolism and enhance mitochondrial function. In addition, melatonin protected MSCs implanted into tissues and organs with ischaemic injuries against senescence, apoptosis and ischaemia-related conditions and promoted injury repair and tissue regeneration. This study confirmed that the enhanced ability of therapeutic cells implanted in injured tissues to cope with stress condns such as ischaemia and itiohypoxia via PINK1 overexpression could further improve IRI-AKI. This research team focused on mitophagy and the PINK1/Parkin pathway, observed the ability of modified BMSCs to repair IRI-AKI, directly transfected PINK1 into BMSCs to reduce the interference of other substances in the pathway, optimized BMSCs and observed the ability of these cells to repair IRI-AKI. The results revealed that PINK1 could enhance BMSC-mediated repair of IRI-AKI, which may be realized by PINK1-mediated enhancement of BMSC resistance to the stress response of RTECs under hypoxic and inflammatory conditions ( Figs. 4–6) and the regulatory effect of mitophagy during IRI-AKI (Fig. 7). The mechanism by which BMSCs repair IRI-AKI is closely related to PINK1. The increased expression level of PINK1 may improve the activity of BMSCs implanted in the AKI microenvironment and achieve favourable therapeutic effects on AKI. Therefore, PINK1 is a potential target for the treatment of IRI-AKI.

However, there are still limitations to the application of stem cell therapy in the clinical environment, such as side effects, high cost, difficulties in controlling the optimal infusion time and the selection of the optimal infusion approach [14]. In addition, BMSCs can damage the lung and liver to a certain extent during kidney repair in mice, which may be caused by cell embolism. According to a study on a rodent kidney injury model, after intravenous injection of BMSCs, most cells were trapped in both lungs due to pulmonary first-pass elimination, which would cause some damage to lung tissues [32]. In addition, a meta-analysis revealed that it is difficult to avoid lung tissue injury resulting from the blockage of BMSCs in both lungs due to kidney injury caused by different methods, the injection of BMSCs at different time points after injury and different methods of BMSC injection [33]. BMSCs overexpressing PINK1 can mitigate lung injury and may promote the entry of BMSCs into damaged organs. Furthermore, BMSCs have a favourable reparative effect on damaged tissues after undergoing through pulmonary first-pass elimination. This effect may be related to the close relationship between the repair mechanism of BMSCs and their paracrine function. Moreover, PINK1 can enhance the paracrine function of BMSCs. BMSCs overexpressing PINK1 have an enhanced ability to promote the repair of kidney tissues damaged by IRI-AKI. In addition, these cells can secrete abundant cytokines and growth factors to promote immunosuppression, inhibit inflammation and anti-apoptosis and promote proliferation, which promote the recovery of renal function. As a cell transplantation strategy, BMSCs overexpressing PINK1 have significant therapeutic potential, therapeutic attraction and clinical application value for the treatment of IRI-AKI. There are also practical problems regarding the use of BMSCs overexpressing PINK1 as a treatment method. For example, reducing pulmonary first-pass elimination without reducing the specific curative effect on IRI-AKI, the level of PINK1 carried by BMSCs, and the verification of the accurate targeting are challenges that should be overcome in the future.

## Conclusion

In this study, PINK1 overexpression enhanced BMSC-mediated repair of IRI-AKI, reduced injured tissue cell apoptosis, reduced T-cell infiltration, increased macrophage infiltration, and improved the inflammatory response. In addition, PINK1 enhanced BMSCs and their resistance to the stress response of RTECs in response to hypoxia and inflammation. In addition, PINK1 regulates mitophagy during IRI-AKI. These findings provide a new direction and target for BMSC-mediated repair of IRI-AKI.

## Data Availability

The datasets used and/or analysed in the current study are available from the corresponding author upon reasonable request.

## Abbreviations

AKI: Acute kidney injury
BMSCs: Bone mesenchymal stromal cells
PINK1: PTEN-induced putative kinase-1
IRI: Ischaemia/reperfusion injury
IRI-AKI: Ischaemia/reperfusion-induced acute kidney injury
RTECs: Renal tubular epithelial cells
LC3: Light chain 3
mTOR: Mammalian target of rapamycin
ROS: Reactive oxygen species
SCR: Serum creatinine
BUN: Blood urea nitrogen
HE: Haematoxylin–eosin
TUNEL: Terminal deoxynucleotidyl transferase dUTP nick-end labelling
FCM: Flow cytometry
IL-10: Interleukin-10
TNF-α: Tumour necrosis factor-α
PBMCs: Peripheral blood mononuclear cells
