# Correction: PTEN-induced kinase 1 enhances the reparative effects of bone marrow mesenchymal stromal cells on mice with renal ischaemia/reperfusion-induced acute kidney injury

**DOI:** 10.1007/s13577-023-00909-3

**Published:** 2023-05-05

**Authors:** Chenyu Lin, Wen Chen, Yong Han, Yujie Sun, Xiaoqiong Zhao, Yuan Yue, Binyu Li, Wenmei Fan, Tao Zhang, Li Xiao

**Affiliations:** 1 Institute of Respiratory and Critical Medicine, Beijing Key Laboratory of Organ Transplantation and Immunology Regulatory, the 8th Medical Centre of Chinese PLA General Hospital, No. 17 Heishan Hu road, Qinglongqiao street, Haidian district, Beijing, 100091 China; 2 Jiamusi University, Jiamusi, China

**Correction: Human Cell (2022) 35:1650–1670** 10.1007/s13577-022-00756-8

In the original publication of the article, “Li Xiao” is the corresponding author and not “Chenyu Lin”.

